# Dietary nutrient intake related to higher grade cervical intraepithelial neoplasia risk: a Chinese population-based study

**DOI:** 10.1186/s12986-020-00521-4

**Published:** 2020-11-30

**Authors:** Zhe Wang, Aimin Yang, Jing Yang, Weihong Zhao, Zhilian Wang, Wei Wang, Jintao Wang, Jinghui Song, Li Li, Weiguo Lv, Dongyan Li, Huiqiang Liu, Chen Wang, Min Hao

**Affiliations:** 1 Department of Obstetrics and Gynecology, Second Hospital of Shanxi Medical University, 382 Wuyi Rd, Taiyuan, 030001 Shanxi China; 2 Hong Kong Institute of Diabetes and Obesity, The Chinese University of Hong Kong, Sha Tin, Hong Kong SAR; 3 Department of Epidemiology, School of Public Health, Shanxi Medical University, Taiyuan, China; 4 Department of Obstetrics and Gynecology, Affiliated Hospital of Inner Mongolia Medical University, Huhhot, China; 5 Department of Obstetrics and Gynecology, Affiliated Tumor Hospital of Guangxi Medical University, Nanning, China; 6 Department of Gynecologic Oncology, Women’s Hospital, School of Medicine, Zhejiang University, Hangzhou, Zhejiang China; 7 Department of Pathology, Second Hospital of Shanxi Medical University, Taiyuan, 030001 Shanxi China

**Keywords:** Nutrient intake, Cervical intraepithelial neoplasia, China, Cross-sectional analysis, Vitamin

## Abstract

**Background:**

Dietary nutrient intake plays a significant role in carcinogenesis. Few studies have investigated the association between dietary nutrient intake and cervical intraepithelial neoplasia (CIN) risk in China.

**Methods:**

Data on 2304 women from an ongoing cohort comprising 40,000 women from China in 2014 were included. Study randomly selected 218 out of 2304 people as subjects during 2019. All participants were surveyed through in-person interviews, physical examinations, and laboratory tests. Clinical data were obtained from physical examinations and laboratory tests. Dietary intakes were assessed using a semiquantitative food frequency questionnaire. Nutrition intakes from 26 food sources were calculated using a comprehensive validated database. Descriptive statistics were used to describe the frequency and proportion, and mean and standard deviation of the demographic characteristics. Characteristics were examined for significant differences, and Pearson chi-square tests were used for categoric variables. Logistic regression was used to obtain odds ratios (ORs) and confidence intervals (CIs) for CIN risk in each nutrient intake quartile relative to that in the highest quartile.

**Results:**

The food frequency questionnaire exhibited acceptable reproducibility and reasonable validity in assessing nutrient intakes among these women. After adjusting for multiple confounders, several dietary nutrients showed significant associations with CIN2+ risk. Low dietary folate intake was associated with the risk of CIN2+ (first versus fourth quartile: OR = 1.55, 95% CI 1.03–2.33). Similar results were also observed for vitamin B6 (OR = 1.63, 95% CI 1.08–2.46), vitamin C (OR = 1.59, 95% CI 1.05–2.42), niacin (OR = 1.65, 95% CI 1.08–2.51), and vitamin K (second versus fourth quartile: OR = 1.60, 95% CI 1.05–2.44).

**Conclusions:**

Low folate; vitamin B6, C, and K; and niacin intakes were associated with CIN2+ risk. Nutrients may influence the development of higher grade CIN and cervical cancer.

*Trial registration* The study was registered in the Chinese Clinical Trial Register (ChiCTR-ROC-15006479) (https://www.chictr.org.cn).

## Background

Cervical cancer has serious implications for women's health, and remains the most common female malignancy in virtually all low-resource countries [1]. Globally, almost 530,000 women experience cervical disease development every year, with an associated mortality of 265,000 [2]. Although human papilloma virus (HPV) vaccination has helped in reducing the incidence or prevalence associated with the disease, country-wide vaccination is difficult in developing countries owing to the associated high expenses [3]. The diversification of preventive measures is required.

Cervical carcinogenesis is related to high-risk HPV (HR-HPV) infection [4]. Recent studies have found that the risk factors of cervical intraepithelial neoplasia (CIN) include not only persistent HPV infection and other well studied risk factors such as socioeconomic status, smoking, age at first intercourse, high parity, and oral contraceptive use [5–13], but also dietary nutrient intake, daily habits, customs, and the presence of vaginitis and an abnormal vaginal pH [14]. The intake of fruits, vegetables, and vitamin-rich foods has the potential to prevent HPV infection [14–16]. Dietary nutrients also act as antagonists for CIN risk [17].

Folate, a water-soluble B vitamin, is essential for the synthesis of nucleotides and DNA hypomethylation [18]. A previous study found no association between dietary folate intake and CIN risk [19]. However, other studies observed conflicting results [20–22]. Vitamin B6 reportedly aids in the regulation of the immune system, which is associated with cancer risk [23]. Studies have reported significant associations between dietary vitamin B6 intake and CIN [20, 24]. Vitamin C intake is associated with cervical disease development [25], and can protect against CIN and invasive squamous carcinoma [15, 17, 26, 27]. Vitamin K has anticancer effects [28, 29], and induces cancer cell apoptosis [30]. Recent studies have also found that niacin can protect against cancer recurrence [31, 32].

Shanxi province is a landlocked and economically backward region. The diet of its residents mainly includes staple food, and their vegetable intake is low. The morbidity associated with cervical cancer in Shanxi is 10 times higher than the national average [33]. The previous study showed that the intake of folate-rich food could reduce cervical cancer risk [34]. The plasma nutrient concentrations among the residents of Shanxi are lower than those among people from other areas [21]. It is unclear whether dietary nutrition intake can affect CIN incidence. Accordingly, the current study initiated a large population-based cervical cancer screening program and a prospective cohort study in the Shanxi CIN Cohort. The current study evaluated the associations of the intakes of dietary nutrients such as folate, vitamin B6, vitamin C, vitamin K and niacin, with CIN risk using categorical analyses.

## Methods

### Study population

The data in this study are based on the the baseline survey of the Shanxi CIN Cohort Study in 2014, which includes 40,000 eligible local women from the Shanxi province, China. The rationale, design, and methods of the Shanxi CIN Cohort Study have been detailed elsewhere [21, 35, 36]. Briefly, the study conducted a free cervical cancer screening for eligible women who were permanent residents of the two counties in Shanxi province during 2014.

A total of 40,000 participants were included. All participants were surveyed using a demographic characteristics-related questionnaire and a Pap test based on liquid-based cytology (LBC). Participants with abnormal Pap test results were examined by colposcopy and histopathology. A total of 2769 women were diagnosed as having atypical squamous cells of undetermined significance (ASC-US) and above, and 78 were excluded (68 refused to participate and 10 showed glandular cell abnormalities). Of the 2691 participants for whom pathologic results were available, 1890 had negative results who with abnormal cytology with currently normal histology. Finally, 564 participants were histologically diagnosed with CIN grade 1 (CIN1), and 237 participants with CIN2 or above (CIN2+). Of the 1890 women with negative pathologic results, 1503 participants were included in the study analysis after the exclusion of 387 women who had not fully completed the three parts of the medical examination, including an in-person interview, a physical examination, and a clinical examination. A sample of 2304 women with a mean age of 49.2 ± 9.1 years was enrolled in the present study, Detailed flowchart of this study have been published elsewhere [36]. All inspections and detections were performed under double-blind conditions. The study was approved by the ethics committee of the Second Hospital, Shanxi Medical University and written informed consent was obtained from the participants.

### Data collection

Several types of data was collected in this study, including questionnaire-related data obtained from in-person interviews, and clinical data from physical examinations, laboratory tests and biospecimen collection. In-person interviews were conducted by trained interviewers using a standardized questionnaire. Demographic information included age; years of education; yearly income; tobacco smoking; age at menarche; menopause status; years of intrauterine device (IUD) use; and sexual activity in the menstrual period. Participants completed a 26-item food frequency questionnaire (FFQ). Clinical data were obtained from physical examinations and laboratory tests, including Pap tests, vaginal pH tests, and cervical biopsies. Data on IUD use, squamous-columnar junction (SCJ) visibility, had gynecologic surgery and vaginitis were also collected. Participants provided biological samples that were stored for future work (blood and cervical tissue specimens).

### Clinical laboratory tests

All Pap tests were performed using the LBC method. At least two cytopathologists from the Second Hospital of Shanxi Medical University evaluated the cytologic results, which were reported using Bethesda System (TBS) 2001 terminology. All abnormal cytology slides were further reviewed for quality control by a senior cytopathologist who was blinded to the previous pathology results if the case was of type ASC-US+ or worse. Gynecology specialists from the Second Hospital of Shanxi Medical University identified patients with an abnormal cervix, and biopsy was performed by colposcopy (SLC-2000 device, Shenzhen Goldway Company) according to a standard protocol ≤ 12 week after the Pap test. Gynecology specialists divided the cervix into quadrants and examined each quadrant. All visually abnormal areas were biopsied, and the quadrants without a visible lesion were biopsied at the SCJ (“random biopsy”). Endocervical curettage was also performed. The cases were classified as negative, CIN1, CIN2, CIN3, or squamous cell carcinoma (SCC). In the end, the pathologists performed a double-blinded observation of the Pap test results after diagnosis based on the cervical biopsy or endocervical curettage tissue specimens. If two pathologists presented different diagnoses, the samples were reviewed by another senior pathologist. The three pathologists reviewed difficult or equivocal cases together to arrive at a consensus on diagnosis.

HPV genotyping by HybriMax was performed using residual Pap test specimens with the HPV GenoArray Test Kit (HybriBio Ltd), and cases were divided into the high-risk HPV infection group and “others” (including low-risk HPV and negative cases). This assay can identify 21 types of HPV, including 15 high-risk types (16, 18, 31, 33, 35, 39, 45, 51, 52, 53, 56, 58, 59, 66, and 68). The low-risk HPV types include types 6, 11, 42, 43, 44, and CP8304, which were identified using the flow-through hybridization technique performed with a TC-96/G/H6 HPV DNA Amplification Analyzer and an HMM-2 fast nucleic acid molecule hybridization instrument (HybriBio Ltd).

Vaginal pH testing was performed with a pH test paper (Marok Darmstadt Germany) with the residual Pap test specimens (2304); vaginal pH values exceeding 4.5 were considered abnormal [37]. Cases were divided into two groups based on the pH results: normal pH group (> 3.8, and < 4.5) and abnormal pH group (≥ 4.5).

### Dietary nutrient assessment

The measurement of individuals’ dietary nutrients intake were calculated based on a food frequency questionnaire (FFQ) obtained from in-person interview. The FFQ in this study was nested in the standardized and structured epidemiological questionnaire of the Shanxin CIN Cohort study. The 24 h dietary recall dietary data were collected by trained interviews who recorded amounts of all the food items. The FFQ was designed based on the China Health and Nutrition Survey (CHNS) [38, 39]. Detailed descriptions of the dietary measurements have been published elsewhere [38, 40]. A total of 26 items food was included in the FFQ, which are main food sources for participants in this study. Based on a Chinese National Nutrition Survey in 2002, ten of 26 items food already account for about 85% of the total dietary intake in Chinese population. The FFQ included 26 food items: wheat flour, soybean, cabbage, egg, oats flour, bean curd, celery, cow milk, buckwheat flour, dried bean curd, spinach, pork liver, rice, broad bean, Chinese chives, sunflower seed, millet, potato, carrot, jujube, maize, mushroom, pumpkin, banana, liquor, and tea.

The FFQ data was analyzed using the US Department of Agriculture’s 1994–1996 Continuing Survey of Food Intakes [41]. The average food intake of individuals (gram/day) was calculated. Each nutrients intakes (grams/milligrams/micrograms per day) were then calculated by multiplying the daily food consumption amount (g per day) by the median nutrition content (g per 100 g/mg per 100 mg/μg per 100 μg of food) of that food. The nutrition values from all other FFQ items were combined to obtain the total daily nutrition values. However, the study did not collect the dietary supplement information in this study because the prevalence of nutrients supplement use is very low in Chinese population [42]. The study estimated the level of each dietary nutrient intake as the following equation: Total daily nutrition value = total amount of each food (g/mg/μg)/(day) * intake value per 100 g, per 100 mg, and per 100 μg. Total nutrition intake (g/mg/μg per day) was determined by calculating the sum of the daily nutrition values.

### Assessing the test–retest reliability and relative validity of the FFQ

Randomly selected 218 out of 2304 people as subjects. The study started from January 2019 and lasted for the subsequent six months. During the study period, three consecutive 24-h studies (24-h) were conducted every three months. The first FFQ was collected during the first 24-h in January 2019. FFQ1 was collected after 3 months in March 2019, and FFQ2 was collected after 6 months in June 2019. The ‘weight estimation (WE)’ method for assessing food consumed for evaluation was estimated by the respondents for the weight of each food they consumed in the previous 24 h [43]. The study design is shown in Additional file 1: Fig. 1. Each participant was asked to provide the name and amount of food consumed during the previous 24 h. If the previous day was a special day, for reasons such as banquets or travel, et al., current study would record food consumption 24 h ago, or choose another day to interview participants by telephone. Subjects were not informed of the results until the night before the interview. Record the amount of food mixed with a plate. According to the definition of food quality standard, recalled food is assigned to the corresponding food group.

For fruits consumed, the subjects were required to select the corresponding pictures representing the different sizes of each fruit and record the corresponding estimated weight (in grams). For commercial projects, such as sliced bread, cakes, packaged biscuits, pies and dumplings, record the unit weight (in grams) of the project. Trained interviewers manage FFQ and 24 h through face-to-face interviews. Immediately check all records and resolve any ambiguities in the subject. During the whole study period, each participant had his own interviewer. Participants who did not satisfactorily complete the FFQs or missed more than one out of the four 24-h were excluded from the analyses. Subjects with implausible energy intakes (< 500 kcal or > 5000 kcal) were also excluded as described by previous studies. Extreme values were examined and excluded. A decision about whether or not to exclude the record from analyses was made according to the original FFQs and/or 24-h [44]. The validity of the FFQ methods was assessed by comparing the nutrient intakes derived from the FFQs. A 3-month FFQ was collected from the same subjects after administration of the first interviewer-administered FFQ.

### Statistical analysis

Descriptive statistics were used to describe the frequency and proportion, and mean and standard deviation of the demographic characteristics. Participants’ characteristics were examined for significant differences with a Pearson’s chi-squared test for categoric variables. A logistic regression model was used to calculate the odds ratios (ORs) and their confidence intervals (CIs) for CIN risk in each nutrient intake quartile relative to that in the highest quartile. Tests for a linear trend across increasing quartiles of nutrient intakes were performed by assigning the medians of each nutrient intake to quartiles treated as continuous variables.

Analyses were adjusted for potential confounders. The first model was unadjusted. Next, adjusted for age (< 30, 30–39, 40–49, 50–59, and > 60 years), years of education (< 6, 7–9, and > 9 years), yearly income (< 10,000, 10,000–30,000, and > 30,000 ¥), tobacco smoking (yes and no), age at menarche (< 13, 13- < 15, 15- < 17, and > 17 years), menopause status (yes and no), IUD use (yes and no), years of IUD use (< 10 and ≥ 10 years), sexual activity in the menstrual period (yes and no), history of undergoing a gynecologic surgery (yes and no), and presence of vaginitis (yes and no). In the final multivariable analysis, added other potential clinical confounders, including high-risk HPV (positive and negative), SCJ visibility (fully visualized and not fully visualized), and vaginal pH (< 4.5 and ≥ 4.5).

The current study performed cross-sectional analyses with three knots (25th, 50th, and 75th percentiles) of the 2304 women to examine the association between log-transformed dietary intake levels and CIN risk. The study did not explore the association between dietary intake and SCC risk because of the limited number of SCC cases (n = 19). Statistical analyses were performed using SAS software version 9.3. All reported *P* values were two-sided, with a significance level of 0.05.

## Results

The characteristics of the participants (n = 2304) are shown in Table 1. The proportions of women with CIN1 were 24.5% (n = 564), with CIN2+ (included CIN 2, CIN 3, and SCC, respectively) were accounted for 10.3% (n = 237) of all the cases. CIN and higher grade disease cases accounted for 34.8% (n = 801) of all the cases. and The median ages of the women without CIN, CIN1, and CIN2+ were 51, 51, and 46 years, respectively. Women with CIN2+ were likelier to be 40–49 years old (*P* < 0.05), experience earlier menarche (*P* < 0.05), and have HPV infection (*P* < 0.05). The number of women with CIN2+ in menopause was also low (*P* < 0.05). However, in terms of education level, yearly income, IUD use, tobacco smoking, vaginal pH, vaginitis, gynecologic surgery, sexual activity in the menstrual period, and SCJ *visibility,* no significant differences were observed across the CIN groups.

**Table 1:** Demographics of 2304 Chinese women by cervical histological examination^a^

Characteristics	Total	Without CIN^b^	CIN1	CIN2+^c^	*P* value^d^
No. of participants (%)	2304 (100.0)	1503 (65.2)	564 (24.5)	237 (10.3)	
Age (years)					
< 30	58 (2.5)	36 (2.4)	19 (3.4)	3 (1.3)	
30–39	284 (12.3)	180 (12.0)	63 (11.1)	41 (17.3)	
40–49	711 (30.9)	441 (29.3)	175 (31.0)	95 (40.1)	0.041
50–59	970 (42.1)	666 (44.3)	231 (41.0)	73 (30.8)	
> 60	281 (12.2)	180 (12.0)	76 (13.5)	25 (10.5)	
Education (years)					
0–6	472 (20.5)	309 (20.6)	117 (20.7)	46 (19.4)	
7–9	996 (43.2)	630 (41.9)	239 (42.4)	127 (53.6)	0.047
> 9	836 (36.3)	564 (37.5)	208 (36.9)	64 (27.0)	
Yearly income^e^ (¥)					
< 10,000	262 (11.4)	173 (11.5)	65 (11.5)	24 (10.1)	
10,000–30,000	1119 (48.6)	738 (49.1)	255 (45.2)	126 (53.2)	0.763
> 30,000	923 (40.1)	592 (39.3)	244 (43.3)	87 (36.7)	
Tobacco smoking	49 (2.1)	29 (1.9)	12 (2.1)	8 (3.4)	0.063
Menopause status	1174 (51.0)	803 (53.4)	293 (52.0)	78 (32.9)	0.001
High-risk HPV^f^					
Positive	755 (32.8)	429 (28.5)	168 (29.8)	158 (66.7)	< 0.001
Negative	1549 (67.2)	1074 (71.5)	396 (70.2)	79 (33.3)	
Age at menarche (years)					
< 13	308 (13.4)	181 (12.0)	93 (16.5)	34 (14.3)	
13–< 15	739 (32.1)	466 (31.0)	180 (32.0)	93 (39.2)	0.028
15–< 17	654 (28.4)	452 (30.1)	133 (23.6)	69 (29.1)	
> 17	603 (26.2)	404 (26.9)	158 (28.0)	41 (17.3)	
IUD^g^ use	1081 (46.9)	735 (48.9)	231 (41.0)	115 (48.5)	0.892
Years of IUD use (years)					
< 10	1632 (70.8)	1037 (69.0)	426 (75.5)	169 (71.3)	0.287
≥ 10	672 (29.2)	466 (31.0)	138 (24.5)	68 (28.7)	
SCJ^h^ *visibility*					
Fully visualized	646 (28.0)	382 (25.4)	185 (32.8)	79 (33.3)	0.114
Not fully visualized	1658 (72.0)	1121 (74.6)	379 (67.2)	158 (66.7)	
Vaginal pH					
< 4.5	514 (22.3)	341 (22.7)	106(18.8)	67 (28.3)	0.228
≥ 4.5	1790 (77.7)	1162 (77.3)	458 (81.2)	170 (71.7)	
Had gynecologic surgery	397 (17.2)	255 (17.0)	107 (19.0)	35 (14.8)	0.647
Had vaginitis	140 (6.1)	82 (5.5)	38 (6.7)	20 (8.4)	0.108
Sexual activity in menstrual period	56 (2.4)	39 (2.6)	9 (1.6)	8 (3.4)	0.549

^a^Data were presented as number (%) of participants

^b^CIN, cervical intraepithelial neoplasia. All CIN status is by histology

^c^CIN2+ included CIN 2, CIN 3 and SCC

^d^*P* values for differences between groups were obtained from the chi-square test for categorical categoric variables

^e^Represent in terms of Chinese Renminbi (RMB)

^f^HPV, human papilloma virus

^g^IUD, intrauterine device

^h^SCJ, squamous-columnar junction

**Fig. 1:**
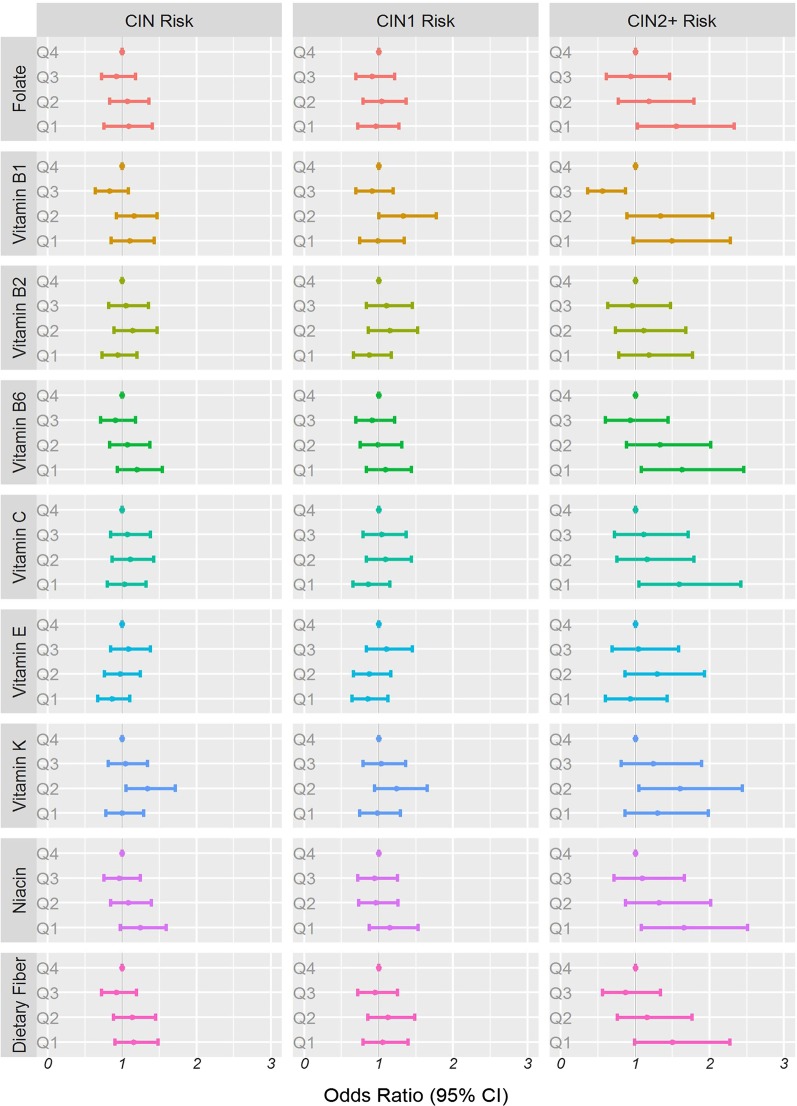
The associations of dietary nutrient intake with the risk of cervical intraepithelial neoplasia, grade 1 cervical intraepithelial neoplasia and grade 2 cervical intraepithelial neoplasia and higher among 2304 women. The vertical lines signify the odds ratios and 95% confidence intervals obtained in the multivariate logistic regression analysis after adjustment for age at the baseline, years of education, income, smoking status, age at menarche, menopause, HPV infection, intrauterine device use, years of intrauterine use, squamous junction status, vaginal pH, sexual activity during menstruation, and history of gynecologic surgery and vaginitis. The horizontal lines denote the quartiles of dietary nutrient intake for dietary folate, dietary vitamin B1, dietary vitamin B2, dietary vitamin B6, dietary vitamin C, dietary vitamin E, dietary vitamin K, dietary niacin, and dietary fiber

Table 2 shows the median dietary nutrient intake in patients without CIN as well as in those with CIN1 and CIN2+. Women with CIN2+ were likelier to have lower dietary nutrient intakes. Figure 1 and Table 3 show the associations between dietary nutrient intake and CIN2+ risk among the 2304 women. Even after complete adjustment, the intakes of most nutrients continued to show significant associations with CIN2+ risk. The study took the fourth quintile as the reference. Compared with the 4th quartile of folate intake, the multivariable-adjusted ORs (95% CI) for CIN2+ risk were 1.55 (1.03–2.33), 1.18 (0.77–1.79), 0.94 (0.61–1.46), 1.00 (reference), respectively.

**Table 2:** Dietary nutrition investigated based on a 26-item food frequency questionnaire among 2304 Chinese women in the Shanxi Cohort Study^a^

Element (intake/day)	Without CIN	CIN1	CIN2+	Total
Folate (μg)	382.8 (298.8–727.2)	383.4 (300.3–804.8)	358.9 (283.8–836.5)	381.0
Vitamin B1 (mg)	1.5 (1.1–2.7)	1.4 (1.2–2.9)	1.3 (1.1–2.6)	1.5
Vitamin B2 (mg)	1.4 (1.1–2.7)	1.4 (1.1–2.8)	1.3 (1.0–2.8)	1.4
Vitamin B6 (mg)	2.1 (1.7–3.8)	2.1 (1.7–4.0)	1.9 (1.6–4.2)	2.1
Vitamin C (mg)	62.9 (45.8–135.9)	63.8 (47.6–143.3)	59.4 (43.2–148.2)	62.8
Vitamin E (mg)	8.6 (4.2–21.8)	9.0 (4.9–21.5)	8.8 (5.4–21.7)	8.8
Vitamin K (μg)	201.2 (127.0–519.4)	197.1 (134.1–569.4)	187.2 (127.7–560.6)	198.2
Niacin (mg)	22.0 (18.1–42.5)	21.7 (17.8–43.3)	21.3 (17.5–42.5)	21.9
Dietary fiber (g)	34.8 (27.5–62.8)	34.4 (27.5–65.8)	32.1 (26.0–62.4)	34.5

^a^Data were presented as median with range

**Table 3:** ORs and 95% Cls for quartiles of dietary nutrients intake with risk of cervical intraepithelial neoplasia grades 2 and above among 2304 women in the study^a^

	Quartiles of dietary nutrients^b^				
	Q1	Q2	Q3	Q4	*P*-trend^c^
Folate					
Median intake (μg/day)	297	381	488	764	
No. of cases	75	57	49	56	
Model 1	1.35 (0.93–1.97)	1.04 (0.70–1.54)	0.85 (0.56–1.28)	1.00 (reference)	< 0.001
Model 2	1.49 (1.01–2.21)	1.08 (0.72–1.62)	0.89 (0.58–1.35)	1.00 (reference)	< 0.001
Model 3	1.55 (1.03–2.33)	1.18 (0.77–1.79)	0.94 (0.61–1.46)	1.00 (reference)	0.008
Vitamin B1					
Median intake (mg/day)	1.1	1.4	1.9	2.7	
No. of cases	72	72	35	58	
Model 1	1.24 (0.84–1.82)	1.41 (0.96–2.06)	0.59 (0.39–0.90)	1.00 (reference)	< 0.001
Model 2	1.29 (0.86–1.93)	1.34 (0.90–1.99)	0.55 (0.36–0.84)	1.00 (reference)	< 0.001
Model 3	1.49 (0.97–2.28)	1.34 (0.89–2.04)	0.56 (0.36–0.87)	1.00 (reference)	0.017
Vitamin B2					
Median intake (mg/day)	1.1	1.4	1.8	2.7	
No. of cases	62	63	51	61	
Model 1	0.95 (0.65–1.39)	1.07 (0.73–1.57)	0.83 (0.56–1.23)	1.00 (reference)	< 0.001
Model 2	1.07 (0.72–1.58)	1.12 (0.75–1.66)	0.92 (0.61–1.38)	1.00 (reference)	< 0.001
Model 3	1.18 (0.78–1.77)	1.11 (0.73–1.68)	0.96 (0.63–1.47)	1.00 (reference)	0.011
Vitamin B6					
Median intake (mg/day)	1.7	2.1	2.6	3.9	
No. of cases	69	65	47	56	
Model 1	1.27 (0.87–1.86)	1.13 (0.77–1.66)	0.83 (0.55–1.25)	1.00 (reference)	< 0.001
Model 2	1.42 (0.96–2.11)	1.18 (0.79–1.75)	0.87 (0.57–1.32)	1.00 (reference)	< 0.001
Model 3	1.63 (1.08–2.46)	1.33 (0.88–2.01)	0.93 (0.60–1.44)	1.00 (reference)	0.002
Vitamin C					
Median intake (mg/day)	46.0	62.8	86.9	138.9	
No. of cases	73	56	57	51	
Model 1	1.44 (0.98–2.11)	1.13 (0.76–1.70)	1.14 (0.76–1.71)	1.00 (reference)	< 0.001
Model 2	1.56 (1.05–2.33)	1.16 (0.76–1.76)	1.15 (0.76–1.75)	1.00 (reference)	< 0.001
Model 3	1.59 (1.05–2.42)	1.16 (0.75–1.79)	1.11 (0.72–1.71)	1.00 (reference)	0.008
Vitamin E					
Median intake (mg/day)	4.4	8.8	10.8	21.7	
No. of cases	50	67	59	61	
Model 1	0.76 (0.51–1.13)	1.03 (0.71–1.50)	1.02 (0.69–1.50)	1.00 (reference)	< 0.001
Model 2	0.92 (0.61–1.40)	1.13 (0.77–1.66)	1.08 (0.72–1.60)	1.00 (reference)	< 0.001
Model 3	0.93 (0.60–1.43)	1.29 (0.86–1.93)	1.04 (0.69–1.58)	1.00 (reference)	0.011
Vitamin K					
Median intake (μg/day)	133.0	194.0	285.0	534.0	
No. of cases	61	71	51	54	
Model 1	1.12 (0.77–1.65)	1.42 (0.96–2.10)	1.01 (0.68–1.50)	1.00 (reference)	< 0.001
Model 2	1.20 (0.81–1.79)	1.53 (1.02–2.29)	1.05 (0.70–1.58)	1.00 (reference)	< 0.001
Model 3	1.30 (0.86–1.98)	1.60 (1.05–2.44)	1.24 (0.81–1.89)	1.00 (reference)	0.009
Niacin					
Median intake (mg/day)	17.7	21.6	27.9	42.8	
No. of cases	68	60	54	55	
Model 1	1.30 (0.88–1.92)	1.08 (0.73–1.59)	1.02 (0.69–1.51)	1.00 (reference)	< 0.001
Model 2	1.40 (0.94–2.09)	1.14 (0.76–1.70)	0.98 (0.65–1.47)	1.00 (reference)	< 0.001
Model 3	1.65 (1.08–2.51)	1.32 (0.87–2.01)	1.09 (0.71–1.66)	1.00 (reference)	0.009
Dietary fiber					
Median intake (g/day)	27.4	34.5	43.7	63.2	
No. of cases	68	64	49	56	
Model 1	1.23 (0.84–1.81)	1.18 (0.80–1.73)	0.84 (0.56–1.26)	1.00 (reference)	< 0.001
Model 2	1.31 (0.88–1.95)	1.18 (0.79–1.76)	0.80 (0.53–1.22)	1.00 (reference)	< 0.001
Model 3	1.50 (0.99–2.27)	1.16 (0.76–1.76)	0.87 (0.56–1.34)	1.00 (reference)	0.009

CIN1, cervical intraepithelial neoplasia grade 1; CI confidence interval

^a^Values are n or ORs (95% CIs) obtained from logistic regression analysis, based on the highest intake group as the reference, unless otherwise indicated

^b^Model 1: odds ratios unadjusted; Model 2: odds ratios adjusted for age, education years, annual family salary, smoker, age at menarche, menopause status, IUD use, years of IUD use, Sexual activity in menstrual period, had gynecologic surgery, had vaginitis; Model 3: additionally odds ratios adjusted for high-risk HPV, SCJ *visibility*, vaginal pH

^c^*P* values for differences between groups were obtained from the Bonferroni correction test for categoric variables

For vitamin B6, compared with the 4th quartile of vitamin B6 intake, the multivariable-adjusted ORs (95% CI) for CIN2+ risk were 1.63 (1.08–2.46), 1.33 (0.88–2.01), 0.93 (0.60–1.44), 1.00 (reference), respectively. The current study observed an inverse association between dietary vitamin C intake and CIN2+ risk in the participants, compared with the 4th quartile of vitamin C intake, the multivariable-adjusted ORs (95% CI) for CIN2+ risk were 1.59 (1.05–2.42), 1.16 (0.75–1.79), 1.11 (0.72–1.71), 1.00 (reference), respectively. Dietary vitamin K intake was significantly associated with CIN2+ risk among the participants, compared with the 4th quartile of vitamin K intake, the multivariable-adjusted ORs (95% CI) for CIN2+ risk were 1.30 (0.86–1.98), 1.60 (1.05–2.44), 1.24 (0.81–1.89), 1.00 (reference), respectively. An inverse statistically significant association was observed between dietary niacin intake and CIN2+ risk. Compared with the 4th quartile of niacin intake, the multivariable-adjusted ORs (95% CI) for CIN2+ risk were 1.65 (1.08–2.51), 1.32 (0.87–2.01), 1.09 (0.71–1.66), 1.00 (reference), respectively.

Additional file 2: Table 1 shows the associations identified between dietary nutrient intake and CIN2+ risk in the 2304 women through the logistic regression analyses. In adjusted mode, demographics, lifestyle habits, and other covariates were adjusted for, and added adjusted for 9 dietary nutrients. Low dietary vitamin C intake was associated with the risk of CIN2+ were 1.89 (1.01–3.55), 1.33 (0.76–2.32), 1.22 (0.74–2.01), 1.00 (reference). Low dietary vitamin K was associated with the risk of CIN2+ were 1.87 (0.97–3.61), 2.18 (1.23–3.86), 1.43 (0.85–2.42), 1.00 (reference). High dietary vitamin B1 was associated with the risk of CIN2+ were 1.08 (0.49–2.41), 0.93 (0.50–1.74), 0.43 (0.24–0.78), 1.00 (reference).

Table 4 shows the associations identified between dietary nutrient intake and CIN1 risk in the 2304 women through the logistic regression analyses. In fully adjusted mode (Fig. 1), demographics, lifestyle habits, and other covariates were adjusted for. The study observed statistically significant associations between dietary vitamin B1 intake and CIN1 risk were 0.99 (0.74–1.34), 1.33 (1.00–1.77), 0.91 (0.69–1.19), 1.00 (reference). However, the current study did not observe statistically significant associations between dietary folate, vitamin B2, vitamin B6, vitamin C, vitamin E, niacin, and dietary fiber with CIN1 risk.

**Table 4:** ORs and 95% Cls for quartiles of dietary nutrients intake with cervical intraepithelial neoplasia grade1 risk among 2304 women in the study^a^

	Quartiles of dietary nutrients^b^				
	Q1	Q2	Q3	Q4	*P*-trend^c^
Folate					
Median intake (μg/day)	297	381	488	764	
No. of cases	132	147	139	146	
Model 1	0.91 (0.69–1.20)	1.01 (0.77–1.32)	0.91 (0.69–1.19)	1.00 (reference)	< 0.001
Model 2	0.95 (0.72–1.26)	1.04 (0.79–1.37)	0.92 (0.70–1.21)	1.00 (reference)	0.012
Model 3	0.96 (0.72–1.27)	1.04 (0.79–1.37)	0.91 (0.69–1.21)	1.00 (reference)	0.020
Vitamin B1					
Median intake (mg/day)	1.1	1.4	1.9	2.7	
No. of cases	134	157	132	141	
Model 1	0.91 (0.68–1.22)	1.25 (0.95–1.65)	0.89 (0.68–1.15)	1.00 (reference)	< 0.001
Model 2	0.98 (0.73–1.31)	1.31 (0.99–1.74)	0.89 (0.68–1.17)	1.00 (reference)	0.011
Model 3	0.99 (0.74–1.34)	1.33 (1.00–1.77)	0.91 (0.69–1.19)	1.00 (reference)	0.017
Vitamin B2					
Median intake (mg/day)	1.1	1.4	1.8	2.7	
No. of cases	123	148	157	136	
Model 1	0.85 (0.64–1.12)	1.13 (0.85–1.48)	1.08 (0.82–1.42)	1.00 (reference)	< 0.001
Model 2	0.88 (0.66–1.17)	1.16 (0.88–1.54)	1.09 (0.83–1.44)	1.00 (reference)	0.011
Model 3	0.87 (0.66–1.17)	1.15 (0.86–1.52)	1.10 (0.83–1.45)	1.00 (reference)	0.020
Vitamin B6					
Median intake (mg/day)	1.7	2.1	2.6	3.9	
No. of cases	144	146	131	143	
Model 1	1.04 (0.79–1.36)	0.99 (0.76–1.30)	0.90 (0.69–1.19)	1.00 (reference)	< 0.001
Model 2	1.08 (0.82–1.43)	1.00 (0.76–1.32)	0.90 (0.68–1.19)	1.00 (reference)	< 0.001
Model 3	1.09 (0.83–1.44)	0.99 (0.75–1.31)	0.91 (0.69–1.21)	1.00 (reference)	0.019
Vitamin C					
Median intake (mg/day)	46.0	62.8	86.9	138.9	
No. of cases	124	149	145	146	
Model 1	0.85 (0.65–1.13)	1.05 (0.81–1.38)	1.01 (0.77–1.33)	1.00 (reference)	< 0.001
Model 2	0.87 (0.65–1.16)	1.09 (0.83–1.44)	1.04 (0.79–1.36)	1.00 (reference)	0.012
Model 3	0.86 (0.65–1.15)	1.09 (0.83–1.44)	1.04 (0.79–1.37)	1.00 (reference)	0.021
Vitamin E					
Median intake (mg/day)	4.4	8.8	10.8	21.7	
No. of cases	131	135	151	147	
Model 1	0.83 (0.63–1.09)	0.86 (0.65–1.13)	1.08 (0.83–1.42)	1.00 (reference)	< 0.001
Model 2	0.84 (0.63–1.11)	0.88 (0.67–1.16)	1.09 (0.83–1.44)	1.00 (reference)	0.013
Model 3	0.85 (0.64–1.12)	0.87 (0.66–1.16)	1.10 (0.83–1.45)	1.00 (reference)	0.023
Vitamin K					
Median intake (μg/day)	133.0	194.0	285.0	534.0	
No. of cases	125	159	139	141	
Model 1	0.94 (0.71–1.23)	1.23 (0.93–1.62)	1.00 (0.77–1.31)	1.00 (reference)	< 0.001
Model 2	0.96 (0.73–1.27)	1.24 (0.94–1.64)	1.01 (0.77–1.33)	1.00 (reference)	0.010
Model 3	0.98 (0.74–1.29)	1.24 (0.94–1.65)	1.03 (0.79–1.36)	1.00 (reference)	0.017
Niacin					
Median intake (mg/day)	17.7	21.6	27.9	42.8	
No. of cases	147	140	135	142	
Model 1	1.11 (0.84–1.46)	0.96 (0.73–1.27)	0.96 (0.73–1.26)	1.00 (reference)	< 0.001
Model 2	1.14 (0.86–1.51)	0.97 (0.74–1.28)	0.93 (0.71–1.23)	1.00 (reference)	0.012
Model 3	1.15 (0.87–1.53)	0.96 (0.73–1.26)	0.94 (0.72–1.25)	1.00 (reference)	0.020
Dietary fiber					
Median intake (g/day)	27.4	34.5	43.7	63.2	
No. of cases	138	148	136	142	
Model 1	0.99 (0.75–1.30)	1.07 (0.82–1.41)	0.92 (0.70–1.21)	1.00 (reference)	< 0.001
Model 2	1.05 (0.79–1.39)	1.12 (0.85–1.47)	0.93 (0.71–1.23)	1.00 (reference)	0.010
Model 3	1.05 (0.79–1.39)	1.12 (0.85–1.48)	0.95 (0.72–1.25)	1.00 (reference)	0.018

CIN1, cervical intraepithelial neoplasia grade 1; CI, confidence interval

^a^Values are n or ORs (95% CIs) obtained from logistic regression analysis, based on the highest intake group as the reference, unless otherwise indicated

^b^Model 1: odds ratios unadjusted; Model 2: odds ratios adjusted for age, education years, annual family salary, smoker, age at menarche, menopause status, IUD use, years of IUD use, Sexual activity in menstrual period, had gynecologic surgery, had vaginitis; Model 3: additionally odds ratios adjusted for high-risk HPV, SCJ *visibility*, vaginal pH

^c^*P* values for differences between groups were obtained from the Bonferroni correction test for categoric variables

The current study also explored the association between each dietary nutrient intake and CIN risk which included CIN1 and CIN2+ (Fig. 1 and Additional file 3: Table 2). Similarly, the study observed the statistical significant associations between folate, vitamin B1, vitamin B2, vitamin B6, vitamin C, vitamin E, niacin, and dietary fiber and risk of CIN, however, non significant associations were observed.

In terms of FFQ validity are shown in Additional file 4: Table 3, The energy-adjusted, and de-attenuated correlation coefficients of the FFQs (FFQ1 and FFQ2) and the 24-h are presented. The energy-adjusted correlation coefficient for WE of FFQ1, r^b^ (0.93–0.99); and the deattenuated coefficient, r^c^ (0.78–0.95) when compared with the 24-h. The energy-adjusted correlation coefficient for WE of FFQ2, r^b^ (0.90–0.99); the deattenuated coefficient, r^c^ (0.45–0.97) when compared with the 24-h, which were a little less than those of FFQ1 versus the 24-h. In the retest reliability of FFQ, the The intra-class correlation coefficient (ICC) of nutrient intake derived from WE FFQs collected at 3-month intervals are shown in Additional file 4: Table 3. The intra-class correlation coefficient for FFQ1 of WE, ICC (0.67–0.94) when compared with the FFQ2. The correlation coefficients were all above 0.6. All nutrients in WE-FFQ have good correlation and agreement.

The current study additionally explored the associations between Age, Age at menarche, High-risk HPV, Menopause status and CIN2+ risk (Additional file 5: Table 4). The results observed the women of the older, the earlier menarche age, the positive HPV infection, not menopause are more likely to be with the CIN2+ risk. In order to explore that tobacco smoking is one of important factors of CIN risk, the study have done the stratified analyses for association between tobacco smoking and the CIN risk (Additional file 6: Table 5). The sutdy also have done the sensitivity analysis of 2255 non-smoking women smokers in the main analysis for association between tobacco smoking and the CIN risk through the logistic regression analyses by adjusting covariates (Additional file 7: Table 6). The study observe statistically significant associations between tobacco smoking and CIN risk.

## Discussion

The current study investigated the association between dietary nutrient intake and CIN risk in this large-scale population-based study. The results indicated that the intakes of several dietary nutrients such as folate; vitamins B6, C, and K; and niacin were associated with CIN2+ risk.

### Multiple micronutrient exposure

Previous epidemiologic studies have supported the role of diet and nutrition in CIN [14], reporting that low intake levels of fruits and vegetables are associated with cervical cancer risk [45]. In our large sample survey, results found that the median intake level of dietary folate, vitamin C, and vitamin E in the CIN2+ group (358.9 μg/d, 59.4 mg/d, and 8.8 mg/d, respectively) was lower than that recommended by the Institute of Medicine (IOM) and the joint committee of the World Health Organization and Food and Agricultural Organization of United Nations (WHO/FAO) (400 μg/d, 75 mg/d, and 15 mg/d, respectively) [46, 47]. These results indicate that dietary nutrient intakes have potential effects on CIN risk after adjustment for multiple factors [48].

### Folate

The role of dietary folate in cervical carcinogenesis is still controversial. Previous epidemiologic (case–control) studies reported that dietary folate intake and CIN2+ risk were not related [49, 50]. However, other studies reported that dietary folate intake was inversely associated with CIN risk [20–22], consistent with our findings. The inconsistencies in the results could be because this study used a large-sample cohort study to obtain results and because the effect of other potential confounders was not evaluated in previous studies. Other factors such as the possibility of inaccurate estimations of food folate composition data or its substantial variations may have also contributed to the same.

Folate plays a role in the de novo synthesis of thymidylate and purines—nucleotides that are required for DNA replication and repair. Folate status affects DNA methylation, both at the gene promoter and genomic levels [51]. Experimental data suggest that the time and dose of folate supplementation during carcinogenesis are important [52]. In this case, the study adjusted for possible risk factors, including the presence of HPV infection, and found that lower dietary folate intake levels were significantly associated with an increasing risk of CIN2+. However, it is still not certain if dietary folate is a pathogenetic factor in CIN.

### Vitamin B6 and vitamin C

The role of the dietary intake of vitamin B6 has been investigated in cancer prevention settings. Pre-studies (case–control design) have demonstrated the protective effects of dietary vitamin B6 intake in CIN2+ [20]. While the results of some studies (cohort and laboratory studies) are consistent with those observed in the present study [24], few studies have focused on the relationship between vitamin B6 and CIN risk. A recent study reported that excessive vitamin B6 supplementation did not have chemo-preventive effects in cancer and may cause harm [53]. These conflicting results could arise because the aforementioned studies focused on cancers other than those of the cervix. Dietary vitamin B6 intake is an inaccurate biomarker for the evaluation of vitamin B6 levels in the human body, and therefore, cannot accurately represent vitamin B6 levels in cells.

The metabolism of vitamin B6 is required for the synthesis of methionine from homocysteine, required for the methylation of DNA, in order to guarantee genomic stability and gene expression [54]. Existing evidence supports the hypothesis that vitamin B6 deficiency may promote cancer development and progression [55].

Dietary vitamin C intake has a significant role in cervical disease [56]. Pre-studies (case–control studies) have shown that dietary vitamin C intake and CIN2+ risk are not related [19, 57]. The findings of recent epidemiologic studies are consistent with those observed in the present study [15, 17, 26, 27]. The inconsistencies in the results may have arisen because effects in pre-studies may be modulated by other dietary factors that the study did not focus on. Although dietary vitamin C intake cannot provide precise information on the vitamin C status in the human body, it could be an appropriate marker for the status of daily vitamin C intake in humans. The conflicting results could have arisen because our study was based on an FFQ, and there was thus a risk of self-report bias, recall bias, or bias arising from assessing food intake based on the amount eaten by other family members. It has been hypothesized that high concentrations of vitamin C may inhibit the GAPDH pathway, leading to an energy crisis in cancer cells [58]. Future research must explore the mechanism of action of such nutrients against cervical cancer.

### Niacin, vitamin K, and vitamin B1

A recent study found that dietary niacin intake had an important role in cancer risk [59]. However, in another study, no association was observed between dietary niacin intake and CIN risk [20]. However, our study results showed that dietary niacin intake was inversely associated with CIN2+ risk. The inconsistencies in the results could be attributed to the inaccurate assessment of niacin deficiency. A previous study did not consider several factors, such as genetic polymorphism. When measurement errors occur independently of outcomes, there is a tendency that results may be biased toward the null hypothesis [60]. The results found that dietary niacin intake was negatively correlated to CIN2+ risk after adjusting for confounders, including the presence of HPV infection. However, the exact mechanisms remain to be elucidated. The dietary status of niacin has the potential to influence DNA repair, genomic stability, and the immune system, eventually impacting cancer risk. The vitamin also participates in a wide variety of ADP-ribosylation reactions [59].

Numerous studies have shown the anticancer effects of vitamin K and thiamine in various cancers [61]. Our results suggest that dietary vitamin K intake could set a range of carcinogenic doses and therefore would be alert to avoid the risk of carcinogenesis in this dose range. This vitamin has protective effects against CIN2+ when the dose is optimal. The underlying mechanism of the relationship between vitamin K intake and CIN risk needs to be further explored. Some studies have found that vitamin B1 has anticancer effects in myeloproliferative neoplasms [62] and oral SCC [63]. Our study results showed that dietary vitamin B1 intake was significantly associated with CIN1 risk. However, it was not significantly associated with CIN2+ risk, and whether vitamin B1 is significantly associated with the risk of cervical cancer needs to be clarified via results derived from studies with accurate information [64, 65].

The study only found vitamin B1 intake to be significantly associated with CIN1 risk. One reason for this could be that CIN1—a low-grade cervical lesion—is highly likely to get resolved. CIN1 presence cannot predict CIN2+ risk. Thus, dietary nutrient intake may be associated with the development of high-grade cervical lesions.

### Limits and strengths

This study has several strengths. First, this population-based study included the largest sample size so far for the evaluation of the association between dietary nutrition intake and CIN risk, ensuring effective power for the detection of statistical significance. Second, the obtained data on the objective assessments of squamous junction types; vaginal pH, HPV types, and CIN-related clinical examinations based on LBC, colposcopy, and colposcopy and cervical biopsy, which may confirm the veracity of the diagnoses from the laboratorial statistics. Third, comprehensive data on potential confounders were also carefully measured and analyzed, minimizing the probability of bias. Finally, the current study analyzed the nature of the dose–response relationships of dietary nutrient intake with CIN1 and CIN2+ risk. However, the study also has some limitations. First, the study had a cross-sectional design, and causality could not be standardized. The FFQ was based on screening and the baseline survey of the Shanxi CIN Cohort Study. As there are only 26 items, the FFQ may be limited to broad categories of foods in which the nutrient values precisely reflect the actual intake, given the potential for wide diversity in the actual foods consumed by individual participants in those categories, as a result, the presence of bias cannot be ruled out [38, 41]. Another limitation was that this study could not evaluate the association between local lifestyle factors that may lead to low dietary intake levels and unhealthy behaviors, and CIN development [66]. Additionally, statistical power of small sample size CIN2+ cases is limited, which may reduces the likelihood that a statistically significant result reflects a true effect. This problem should be addressed with an increasing requirement for strong statistical evidence in the future study.

## Conclusions

In summary, the intakes of dietary nutrients such as folate; vitamins B6, C, and K; and niacin were associated with CIN2+ risk, supporting the hypothesis that nutrients play a role in the development of higher grade CIN and cervical cancer. Provide dietary measures to preventing CIN.

## Supplementary information

**Additional file 1: Fig. 1.** Design of the reproducibility and validation study. FFQ1 was administered during the first 24-h and FFQ2 was administered during the last 24-h. The four 24-h were administered at intervals of three months.

**Additional file 2: Table 1.** ORs and 95% Cls for quartiles of dietary nutrients intake with with risk of cervical intraepithelial neoplasia grades 2 and above among 2304 women in the study.

**Additional file 3: Table 2.** ORs and 95% Cls for quartiles of dietary nutrients intake with cervical intraepithelial neoplasia risk among 2304 women in the study.

**Additional file 4: Table 3.** Reliability and validity of food-frequency questionnaires^a^ (24-h, FFQ1 and FFQ2) in 218 women in China.

**Additional file 5: Table 4.** ORs and 95% CIs for the associations between Age, Age at menarche, Menopause status and high-risk HPV infection with the risk of cervical intraepithelial neoplasia grades 2 and above.

**Additional file 6: Table 5.** ORs and 95% CIs for the associations between tobacco smoking with the risk of cervical intraepithelial neoplasia among 2304 women in the study.

**Additional file 7: Table 6.** ORs and 95% Cls for quartiles of dietary nutrients intake with risk of cervical intraepithelial neoplasia grades 2 and above among participants among 2255 non-smoking women in the study.

## Data Availability

The datasets generated and/or analyzed during the current study are not publicly available due to privacy protection of the participants but are available from the corresponding author on reasonable request.

## Abbreviations

ASC-US: Atypical squamous cells of undetermined significance
CIN: Cervical intraepithelial neoplasia
CIN1: Cervical intraepithelial neoplasia grade 1
CIN2: Cervical intraepithelial neoplasia grade 2
CIN3: Cervical intraepithelial neoplasia grade 3
ECC: Endocervical curettage
HPV: Human papillomavirus
LBC: Liquid based cytology
SCC: Squamous cell carcinoma
FFQ: Food frequency questionnaire
IUD: Intrauterine device
SCJ: Squamous-columnar junction
TBS: The Bethesda system
