# Supplementary figures and images for: Dietary nutrient intake related to higher grade cervical intraepithelial neoplasia risk: a Chinese population-based study
Source: Nutr Metab (Lond). 2020 Nov 30;17:100. doi: 10.1186/s12986-020-00521-4 (PMC7708219; doi:10.1186/s12986-020-00521-4)

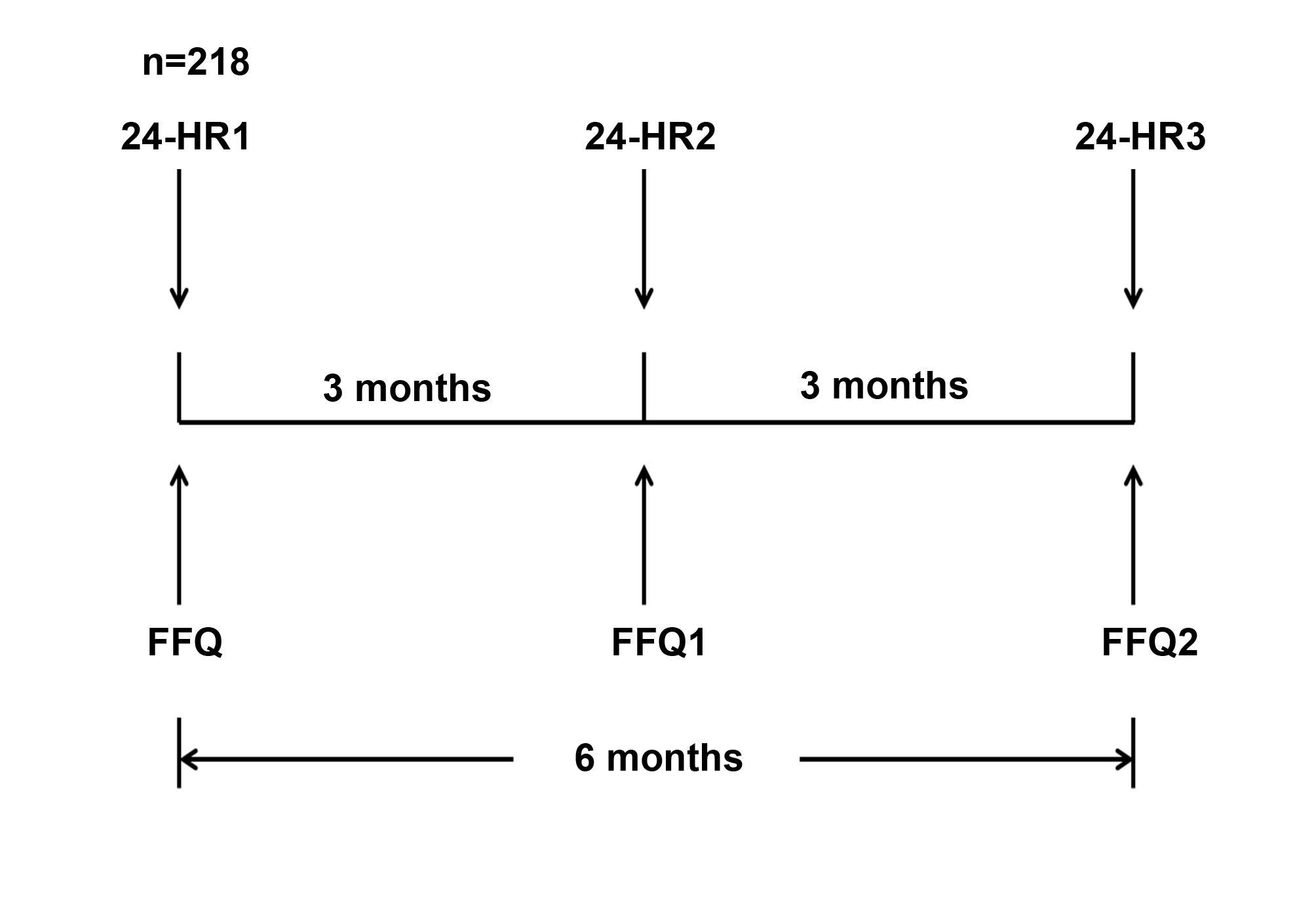

Supplement: Supplementary file 1 — Additional file 1: Fig. 1. Design of the reproducibility and validation study. FFQ1 was administered during the first 24-h and FFQ2 was administered during the last 24-h. The four 24-h were administered at intervals of three months. [file 12986_2020_521_MOESM1_ESM.tif]
