# Supplementary material for: Dietary nutrient intake related to higher grade cervical intraepithelial neoplasia risk: a Chinese population-based study
Source: Nutr Metab (Lond). 2020 Nov 30;17:100. doi: 10.1186/s12986-020-00521-4 (PMC7708219; doi:10.1186/s12986-020-00521-4)
Supplement: Supplementary file 2 — Additional file 2: Table 1. ORs and 95% Cls for quartiles of dietary nutrients intake with with risk of cervical intraepithelial neoplasia grades 2 and above among 2304 women in the study. [file 12986_2020_521_MOESM2_ESM.docx]

**Supplemental Table 1** ORs and 95% Cls for quartiles of dietary nutrients intake with with risk of cervical intraepithelial neoplasia grades 2 and above among 2,304 women in the study^a^

| **Quartiles of dietary nutrients**^b^ | | | | | |
| --- | --- | --- | --- | --- | --- |
|  | **Q1** | **Q2** | **Q3** | **Q4** | ***P*-trend**^c^ |
| **Folate** | | | | | |
| Median intake, μg/day | 297 | 381 | 488 | 764 |  |
| No. of cases | 75 | 57 | 49 | 56 |  |
| Model 1 | 1.35 (0.93-1.97) | 1.04 (0.70-1.54) | 0.85 (0.56-1.28) | 1.00 (reference) | <0.001 |
| Model 2 | 2.13 (1.06-4.27) | 1.48 (0.81-2.68) | 1.11 (0.66-1.88) | 1.00 (reference) | <0.001 |
| Model 3 | 1.94 (0.92-4.08) | 1.38 (0.73-2.61) | 1.08 (0.62-1.88) | 1.00 (reference) | 0.001 |
| **Vitamin B1** |  | | | | |
| Median intake, mg/day | 1.1 | 1.4 | 1.9 | 2.7 |  |
| No. of cases | 72 | 72 | 35 | 58 |  |
| Model 1 | 1.24 (0.84-1.82) | 1.41 (0.96-2.06) | 0.59 (0.39-0.90) | 1.00 (reference) | <0.001 |
| Model 2 | 0.90 (0.41-1.98) | 0.98 (0.53-1.80) | 0.46 (0.26-0.82) | 1.00 (reference) | <0.001 |
| Model 3 | 1.08 (0.49-2.41) | 0.93 (0.50-1.74) | 0.43 (0.24-0.78) | 1.00 (reference) | 0.004 |
| **Vitamin B2** |  | | | | |
| Median intake, mg/day | 1.1 | 1.4 | 1.8 | 2.7 |  |
| No. of cases | 62 | 63 | 51 | 61 |  |
| Model 1 | 0.95 (0.65-1.39) | 1.07 (0.73-1.57) | 0.83 (0.56-1.23) | 1.00 (reference) | <0.001 |
| Model 2 | 0.78 (0.42-1.44) | 0.94 (0.55-1.60) | 0.77 (0.48-1.23) | 1.00 (reference) | <0.001 |
| Model 3 | 0.85 (0.44-1.64) | 0.89 (0.50-1.57) | 0.83 (0.50-1.37) | 1.00 (reference) | 0.006 |
| **Vitamin B6** |  | | | | |
| Median intake, mg/day | 1.7 | 2.1 | 2.6 | 3.9 |  |
| No. of cases | 69 | 65 | 47 | 56 |  |
| Model 1 | 1.27 (0.87-1.86) | 1.13 (0.77-1.66) | 0.83 (0.55-1.25) | 1.00 (reference) | <0.001 |
| Model 2 | 1.37 (0.65-2.88) | 1.22 (0.66-2.25) | 0.91 (0.53-1.55) | 1.00 (reference) | <0.001 |
| Model 3 | 2.01 (0.94-4.29) | 1.50 (0.79-2.83) | 1.04 (0.59-1.82) | 1.00 (reference) | 0.001 |
| **Vitamin C** |  | | | | |
| Median intake, mg/day | 46.0 | 62.8 | 86.9 | 138.9 |  |
| No. of cases | 73 | 56 | 57 | 51 |  |
| Model 1 | 1.44 (0.98-2.11) | 1.13 (0.76-1.70) | 1.14 (0.76-1.71) | 1.00 (reference) | <0.001 |
| Model 2 | 2.04 (1.14-3.66) | 1.45 (0.86-2.45) | 1.38 (0.86-2.20) | 1.00 (reference) | <0.001 |
| Model 3 | 1.89 (1.01-3.55) | 1.33 (0.76-2.32) | 1.22 (0.74-2.01) | 1.00 (reference) | 0.001 |
| **Vitamin E** |  | | | | |
| Median intake, mg/day | 4.4 | 8.8 | 10.8 | 21.7 |  |
| No. of cases | 50 | 67 | 59 | 61 |  |
| Model 1 | 0.76 (0.51-1.13) | 1.03 (0.71-1.50) | 1.02 (0.69-1.50) | 1.00 (reference) | <0.001 |
| Model 2 | 0.61 (0.35-1.08) | 0.86 (0.53-1.39) | 0.91 (0.58-1.44) | 1.00 (reference) | <0.001 |
| Model 3 | 0.67 (0.36-1.24) | 1.01 (0.60-1.71) | 0.86 (0.53-1.41) | 1.00 (reference) | 0.007 |
| (*Continued*) | | | | | |

| **Supplemental Table 1** *Continued* | | | | | |
| --- | --- | --- | --- | --- | --- |
| **Quartiles of dietary nutrients**^b^ | | | | | |
|  | **Q1** | **Q2** | **Q3** | **Q4** | ***P*-trend**^c^ |
| **Vitamin K** |  |  |  |  |  |
| Median intake, μg/day | 130.0 | 198.2 | 289.5 | 534.0 |  |
| No. of cases | 61 | 70 | 52 | 54 |  |
| Model 1 | 1.08 (0.72-1.61) | 1.46 (0.99-2.15) | 0.97 (0.64-1.46) | 1.00 (reference) | <0.001 |
| Model 2 | 1.51 (0.82-2.79) | 1.83 (1.08-3.13) | 1.12 (0.69-1.82) | 1.00 (reference) | <0.001 |
| Model 3 | 1.87 (0.97-3.61) | 2.18 (1.23-3.86） | 1.43 (0.85-2.42) | 1.00 (reference) | 0.001 |
| **Niacin** |  | | | | |
| Median intake, mg/day | 17.7 | 21.6 | 27.9 | 42.8 |  |
| No. of cases | 68 | 60 | 54 | 55 |  |
| Model 1 | 1.30 (0.88-1.92) | 1.08 (0.73-1.59) | 1.02 (0.69-1.51) | 1.00 (reference) | <0.001 |
| Model 2 | 1.32 (0.70-2.50) | 1.10 (0.63-1.91) | 0.99 (0.61-1.62) | 1.00 (reference) | <0.001 |
| Model 3 | 1.66 (0.86-3.22) | 1.35 (0.76-2.41) | 1.03 (0.61-1.72) | 1.00 (reference) | 0.001 |
| **Dietary Fiber** |  | | | | |
| Median intake, g/day | 27.4 | 34.5 | 43.7 | 63.2 |  |
| No. of cases | 68 | 64 | 49 | 56 |  |
| Model 1 | 1.23 (0.84-1.81) | 1.18 (0.80-1.73) | 0.84 (0.56-1.26) | 1.00 (reference) | <0.001 |
| Model 2 | 0.76 (0.34-1.71) | 0.84 (0.44-1.60) | 0.68 (0.39-1.16) | 1.00 (reference) | 0.001 |
| Model 3 | 0.86 (0.36-2.07) | 0.78 (0.39-1.58) | 0.66 (0.37-1.18) | 1.00 (reference) | 0.007 |

^a^: Values are n or ORs (95% CIs) obtained from logistic regression analysis, based on the highest intake group as the reference, unless otherwise indicated.

Abbreviations: CIN= cervical intraepithelial neoplasia, CI= confidence interval.

^b^: Model 1: odds ratios unadjusted;

Model 2: odds ratios adjusted for Dietary Folate, Vitamin B1, Vitamin B2, Vitamin B6, Vitamin C, Vitamin E, Vitamin K, Niacin and Dietary Fiber;

Model 3: additionally odds ratios adjusted for age, education years, annual family salary, smoker, age at menarche, menopause status, IUD use, years of IUD use, Sexual activity in menstrual period, had gynecologic surgery, had vaginitis, high-risk HPV, SCJ visibility, vaginal pH.

^c^: *P* values for differences between groups were obtained from the Bonferroni correction test for categoric variables.
