# Supplementary material for: Dietary nutrient intake related to higher grade cervical intraepithelial neoplasia risk: a Chinese population-based study
Source: Nutr Metab (Lond). 2020 Nov 30;17:100. doi: 10.1186/s12986-020-00521-4 (PMC7708219; doi:10.1186/s12986-020-00521-4)
Supplement: Supplementary file 4 — Additional file 4: Table 3. Reliability and validity of food-frequency questionnairesa (24-h, FFQ1 and FFQ2) in 218 women in China. [file 12986_2020_521_MOESM4_ESM.docx]

**Supplemental Table 3.** Reliability and validity of food-frequency questionnaires^a^ (24-HRs，FFQ1 and FFQ2) in 218 women in China.

| **Variables** | **FFQ1 vs. 24-HRs** | | **FFQ2 vs. 24-HRs** | | **FFQ1 vs. FFQ2** | |
| --- | --- | --- | --- | --- | --- | --- |
| Food elements | r-Energy adjusted^b^ | r-Deattenuated^c^ | r-Energy adjusted^b^ | r-Deattenuated^c^ | ICC^d^ | *P* value^e^ |
| Folate (μg) | 0.99 | 0.94 | 0.99 | 0.45 | 0.67 | <0.001^*^ |
| Vitamin B1 (mg) | 0.91 | 0.91 | 0.95 | 0.93 | 0.89 | <0.001^*^ |
| Vitamin B6 (mg) | 0.96 | 0.95 | 0.97 | 0.97 | 0.90 | <0.001^*^ |
| Vitamin C (mg) | 0.95 | 0.89 | 0.90 | 0.83 | 0.92 | <0.001^*^ |
| Vitamin K (μg) | 0.99 | 0.78 | 0.98 | 0.79 | 0.94 | <0.001^*^ |
| Niacin (mg) | 0.93 | 0.94 | 0.95 | 0.96 | 0.89 | <0.001^*^ |
| Energy (Kcal) |  |  |  |  | 0.90 | <0.001^*^ |

^a^: Data were log-transformed.

^b^: Data were log-transformed and energy-adjusted correlation coefficients.

^c^: Data were log-transformed and de-attenuated correlation coefficients.

^d^: Data were log-transformed and intra-class correlation coefficients.

^e^: *P* value of intraclass correlation coefficients between two FFQ administrations. ^*^Significant estimates (P<0.05).
