# Supplementary material for: Dietary nutrient intake related to higher grade cervical intraepithelial neoplasia risk: a Chinese population-based study
Source: Nutr Metab (Lond). 2020 Nov 30;17:100. doi: 10.1186/s12986-020-00521-4 (PMC7708219; doi:10.1186/s12986-020-00521-4)
Supplement: Supplementary file 5 — Additional file 5: Table 4. ORs and 95% CIs for the associations between Age, Age at menarche, Menopause status and high-risk HPV infection with the risk of cervical intraepithelial neoplasia grades 2 and above. [file 12986_2020_521_MOESM5_ESM.docx]

**Supplemental Table 4** ORs and 95% CIs for the associations between Age, Age at menarche, Menopause status and high-risk HPV infection with the risk of cervical intraepithelial neoplasia grades 2 and above^a^

|  | Participants, n | | ORs (95% CIs)^b^ | | |
| --- | --- | --- | --- | --- | --- |
|  | Normal | Case | Model 1 | Model 2 | Model 3 |
| CIN2+ |  |  |  |  |  |
| Age, y |  |  |  |  |  |
| Q5 (>60) | 180 | 25 | 1.00 (reference) | 1.00 (reference) | 1.00 (reference) |
| Q4 (50-59) | 666 | 73 | 0.79 (0.49-1.28) | 0.79 (0.49-1.29) | 0.62 (0.36-1.08) |
| Q3 (40-49) | 441 | 95 | 1.55 (0.97-2.49) | 1.48 (0.91-2.39) | 0.50 (0.25-1.00) |
| Q2 (30-39) | 180 | 41 | 1.64 (0.96-2.81) | 1.54 (0.89-2.66) | 0.40 (0.18-0.87) |
| Q1 (<30) | 36 | 3 | 0.60 (0.17-2.09) | 0.59 (0.17-2.08) | 0.16 (0.04-0.67) |
| Age at menarche, y |  |  |  |  |  |
| Q4 (>17) | 404 | 41 | 1.00 (reference) | 1.00 (reference) | 1.00 (reference) |
| Q3 (15-<17) | 452 | 69 | 1.50 (1.00-2.27) | 1.46 (0.97-2.20) | 1.49 (0.96-2.30) |
| Q2 (13-<15) | 466 | 93 | 1.97 (1.33-2.91) | 1.93 (1.30-2.85) | 1.79 (1.17-2.73) |
| Q1 (<13) | 181 | 34 | 1.85 (1.14-3.01) | 1.87 (1.14-3.08) | 1.95 (1.15-3.32) |
| High-risk HPV |  |  |  |  |  |
| Positive | 429 | 158 | 1.00 (reference) | 1.00 (reference) | 1.00 (reference) |
| Negative | 1074 | 79 | 0.20 (0.15-0.27) | 0.20 (0.15-0.27) | 0.22 (0.16-0.30) |
| Menopause status |  |  |  |  |  |
| Positive | 803 | 78 | 1.00 (reference) | 1.00 (reference) | 1.00 (reference) |
| Negative | 700 | 159 | 2.34 (1.75-3.12) | 2.27 (1.69-3.04) | 2.24 (1.37-3.67) |

^a^: Values are n or ORs (95% CIs) obtained from logistic regression analysis, using the highest intake group as the reference, unless otherwise indicated.

CIN, cervical intraepithelial neoplasia; HPV, human papillomavirus; Q, quartile; y, year.

^b^: Model 1: unadjusted. Model 2: adjusted for education years, annual family salary, smoker, IUD use, years of IUD use, Sexual activity in menstrual period, had gynecologic surgery, had vaginitis. Model 3: odds ratios adjusted for SCJ visibility, vaginal pH. additionally, Age was adjusted for Age at menarche, high-risk HPV, and Menopause status; Age at menarche was adjusted for Age, high-risk HPV, and Menopause status, high-risk HPV was adjusted for Age, Age at menarche, and Menopause status; Menopause status was adjusted Age, Age at menarche, and high-risk HPV.
