# Supplementary material for: Dietary nutrient intake related to higher grade cervical intraepithelial neoplasia risk: a Chinese population-based study
Source: Nutr Metab (Lond). 2020 Nov 30;17:100. doi: 10.1186/s12986-020-00521-4 (PMC7708219; doi:10.1186/s12986-020-00521-4)
Supplement: Supplementary file 6 — Additional file 6: Table 5. ORs and 95% CIs for the associations between tobacco smoking with the risk of cervical intraepithelial neoplasia among 2304 women in the study. [file 12986_2020_521_MOESM6_ESM.docx]

**Supplemental Table 5** ORs and 95% CIs for the associations between tobacco smoking with the risk of cervical intraepithelial neoplasia among 2,304 women in the study ^a^

|  | Participants, n | | ORs (95% CIs)^b^ | | |
| --- | --- | --- | --- | --- | --- |
|  | Normal | Case | Model 1 | Model 2 | Model 3 |
| CIN2+ |  |  |  |  |  |
| Tobacco smoking |  |  |  |  |  |
| Yes | 49 | 8 | 1.00 (reference) | 1.00 (reference) | 1.00 (reference) |
| No | 2255 | 229 | 0.56 (0.25-1.25) | 0.50 (0.23-1.12) | 0.41 (0.17-0.99) |
| CIN1  Tobacco smoking |  |  |  |  |  |
| Yes | 49 | 12 | 1.00 (reference) | 1.00 (reference) | 1.00 (reference) |
| No | 2255 | 552 | 0.91 (0.46-1.79) | 0.86 (0.43-1.70) | 0.95 (0.47-1.93) |
|  |  |  |  |  |  |

^a^: Values are n or ORs (95% CIs) obtained from logistic regression analysis, using the highest intake group as the reference, unless otherwise indicated.

CIN, cervical intraepithelial neoplasia.

^b^: Model 1: unadjusted. Model 2: adjusted for Dietary Folate, Vitamin B1, Vitamin B2, Vitamin B6, Vitamin C, Vitamin E, Vitamin K, Niacin and Dietary Fiber. Model 3: odds ratios adjusted for Age, Age at menarche, high-risk HPV, Menopause status, education years, annual family salary, IUD use, years of IUD use, Sexual activity in menstrual period, had gynecologic surgery, had vaginitis.SCJ visibility, vaginal pH.
